# Piperacillin-Tazobactam-Induced Urticaria and Angioedema: A Case Report With Literature Review

**DOI:** 10.7759/cureus.58877

**Published:** 2024-04-23

**Authors:** Sajal Pandya, Chetna Patel, Brijesh Sojitra, Nilkanth Chaudhari

**Affiliations:** 1 Pharmacology, Government Medical College, Surat, IND; 2 Pharmacology, Government Medical College and New Civil Hospital, Surat, IND; 3 Pharmacology and Therapeutics, Government Medical College, Surat, IND; 4 Pulmonary Medicine, Government Medical College, Surat, IND

**Keywords:** adherence, adverse drug reaction, angioedema, urticaria, piperacillin-tazobactam

## Abstract

Drug-induced urticaria and angioedema cases are typically reversible upon discontinuation and can be triggered by antibiotics, angiotensin-converting enzyme inhibitors, or nonsteroidal anti-inflammatory drugs. Piperacillin-tazobactam, a common broad-spectrum antimicrobial, has been linked to severe adverse reactions, such as thrombocytopenia, hemolytic anemia, and Steven Johnson syndrome in some cases. A 35-year-old male presented to the emergency department with fever, cough, and acute breathlessness, complicating his ongoing treatment for pulmonary tuberculosis with bedaquiline and delamanid. He was admitted and received supportive care. On the third day of intravenous piperacillin-tazobactam, he developed drug-induced urticaria and angioedema, which resolved upon discontinuing the drug. Piperacillin/tazobactam-induced hypersensitivity reaction is an immunologic and IgE-mediated immediate reaction. IgE-mediated immediate reactions to three major phenotypes of allergic patients with confirmed to piperacillin/tazobactam are either (1) sensitized to the β-lactam ring or (2) sensitized to the lateral chain of aminopenicillins or (3) selective to piperacillin/tazobactam alone. A skin patch test is advised, or prescribed to avoid hypersensitivity reactions due to piperacillin/tazobactam. This case underscores the challenges of non-adherence to anti-tubercular therapy, leading to drug resistance and prolonged, costly, and sometimes intolerable treatments. Regular patient follow-up, counseling, monitoring, and healthcare provider involvement are essential to enhance treatment adherence. Adverse drug reactions must be promptly reported and managed, and patient-centric approaches are crucial. Digital patient records and standardized data collection are recommended for program evaluation and global policy development. Causality assessment for piperacillin-tazobactam was diagnosed as the probable cause of drug-induced urticaria and angioedema. This case highlights the importance of adherence to tuberculosis treatment to prevent drug resistance. Overall, patient-centered care, monitoring adverse events of drug added, and better data collection are crucial for successful tuberculosis management.

## Introduction

Urticaria is a vascular reaction of the skin consisting of a usually pruritic discrete edematous area of skin [1]. Angioedema presents as non-pitting edema or localized area of soft tissue swelling involving the deeper subcutaneous, submucosal tissues [1,2]. In 49% of patients, urticaria and angioedema occur together. It may be brief and self-limited most of the time, of unknown etiology, allergic, hereditary, and can be a part of the anaphylaxis or immediately after drug administration [1]. Drug-induced urticaria and angioedema are acute onset and reversible on drug withdrawal. It is common with beta-lactam antibiotics, renin-angiotensin-aldosterone system inhibitors, and nonsteroidal anti-inflammatory drugs [3]. Prevalence of beta-lactam-induced hypersensitivity/allergic reactions ranges from 1% to >10% [4]. There is a partial cross-sensitivity between different penicillins. If someone has shown an immediate hypersensitivity reaction to one type of penicillin, it is generally advised not to administer any other type of penicillin to them. There could be up to a 10% chance of cross-reactivity between penicillin derivatives, cephalosporins, and carbapenems. This was attributed to the shared structure known as the ß-lactam ring among these antibiotics [5].

In clinical practice, piperacillin-tazobactam is commonly used as a broad-spectrum antibiotic. Different life-threatening adverse drug reactions (ADRs) like thrombocytopenia in liver-transplant patients, drug-induced hemolytic anemia in neonates, and Steven Johnson syndrome have been reported with long-term use of piperacillin-tazobactam [6-8].

## Case presentation

The patient was a 35-year-old male who came to the emergency outpatient department with complaints of fever and cough for two days and suffering from breathlessness for the last few hours. The patient was a clinically failure case of pulmonary tuberculosis (TB) and started treatment with a bedaquiline and delamanid-based longer oral regimen. He was admitted to the isolation ward and proper oxygen support, bronchodilators, nebulization, antibiotics, intravenous fluid, anti-emetic, diet coverage, and anti-tubercular therapy were given.

Five years back, when the patient was diagnosed with pulmonary TB, he was taking anti-tubercular therapy for five months and discontinued therapy because of skin rashes. In between he was diagnosed with a defaulter case of pulmonary TB and took a drug-resistant treatment regimen for two months. Then again, he was diagnosed with a clinical failure mono H (isoniazid)-resistant TB, and he had taken treatment with a bedaquiline and delamanid-based regimen.

The patient had a medical history of a cor-pulmonary and acute case of typhoid. On examination, the patient was conscious, cooperative, and well-oriented to time, place, and person. The patient was under continuous monitoring of temperature, pulse, respiratory rate, blood pressure, and SpO_2_ (saturation of peripheral oxygen). The basic laboratory investigations for this patient were done (Table 1). A respiratory examination showed the presence of crepitus on the lower left side of the lung.

**Table 1:** Laboratory investigations of the patient

Investigation	Patient’s value	Reference range	Unit
Hemoglobin	9.4	14.0-17	g/dl
Total count	15,700	4000-11,000	/mm^3^
Platelet count	4,97,000	150,000-450,000	/mm^3^
PSMP *Plasmodium falciparum* and *Plasmodium vivax*	Negative		
Random blood sugar	93	70-110	mg/dl
Albumin	3.6	3.5-5.5	g/dl
Bilirubin			
Direct	0.1	0.0-0.3	mg/dl
Indirect	0.2	0.2-0.8	mg/dl
Total	0.3	0.3-1.0	mg/dl
Serum creatinine	0.69	0.74-1.35	mg/dl
Serum potassium	4.2	3.5-5.5	mEq/l
Serum sodium	132	135 and 145	mEq/l
Total protein	6.47	6-8	g/dl
C-reactive protein	106	0.3-1.0	mg/dl
Lactate dehydrogenase (LDH)	423	135-225	u/l
Prothrombin time	15.4	10-13	s
International normalized ratio	1.11	1.1 or below	
HIV	Non-reactive		
Dengue-IgG	Negative		
Dengue-IgM	Negative		
NS-1 antigen	Negative		
Typhi DOT IgM	Positive		
Typhi DOT IgG	Negative		
Microscopic examination	Gram-positive cocci present		

**Figure 1:**
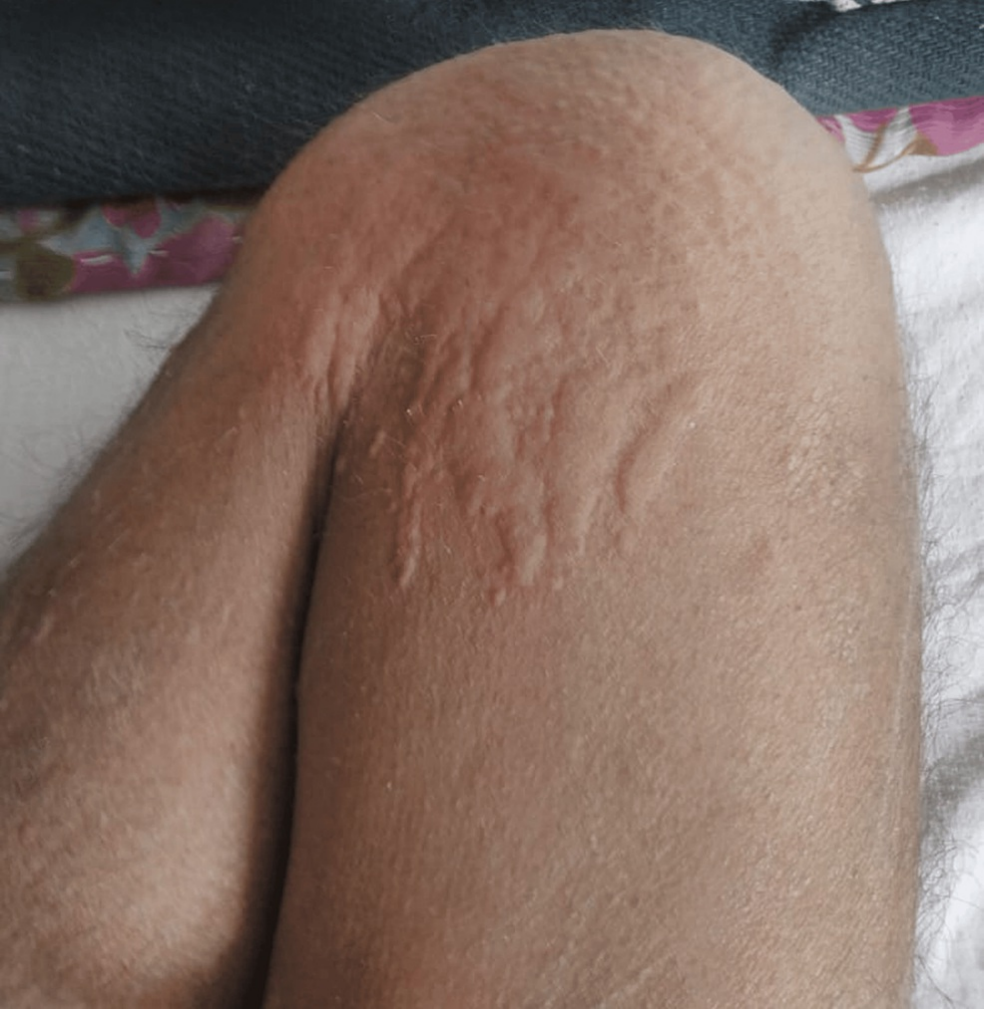
Multiple red elevated erythematous lesions developed on the thigh and the knee due to piperacillin-tazobactam

On the third day of intravenous administration of injection piperacillin-tazobactam 4.5 g every 8 hours, he developed skin lesions over the body, multiple red elevated erythematous lesions with severe itching over the body. Swelling of lips and periorbital area was there. After holding the injection of piperacillin-tazobactam, the patient recovered. The patient was diagnosed with drug-induced acute urticaria and angioedema (Figure 1). The dermatologist prescribed montelukast 10 mg once a day and calamine lotion and advised to avoid piperacillin-tazobactam and other drugs listed to avoid such ADR.

**Figure 2:**
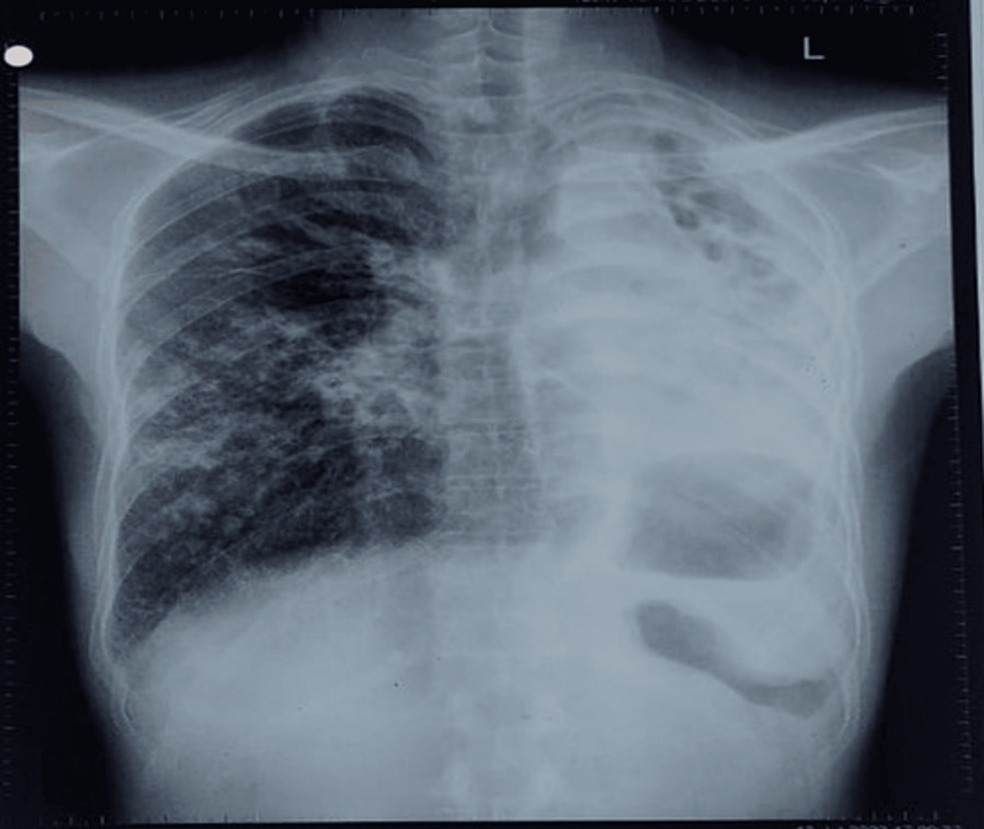
The left side of the lung shows distension and the right side of the lung shows infiltration

Chest X-ray revealed left lung distension and infiltration in the right lung (Figure 2). Ultrasonography of the thorax indicated minimal non-tippable fluid with underlying consolidated lung observed along the left costophrenic angle. The right costophrenic angles appeared clear. There was no evidence of pericardial effusion.

## Discussion

As per WHO-UMC criteria, a temporal relationship between the drug and adverse reaction was present, and de-challenge positive. Causality assessment for piperacillin-tazobactam diagnosed the probable cause of drug-induced urticaria and angioedema. Re-challenge was not performed because of ethical considerations [9]. Assessment according to Naranjo’s scale was 5, which indicates probable result for this ADR [10]. 

In the present case, physicians suspect that the erythematous skin lesions over the body with fever may be due to the dengue virus, typhoid, or drug-induced. Such types of case reports will be helpful to physicians in decision-making for various causes of a single diagnosis. It recommends that drug-induced cases will only be managed by holding the drug, and there is no requirement for laboratory investigations which may be cost-effective.

The present case represents one of the cases of non-adherence to anti-tubercular therapy because of ADRs. After that, sensitive pulmonary TB patients might be converted into drug-resistant TB patients. That can further lead to a long duration of treatment, intolerance, severe adverse effects, and cost burden to patients and the healthcare system. It will also affect the family members, and the disability of the patient will increase and the quality of life will decrease.

According to NTEP guidelines, it is recommended to maintain regular patient follow-up to ensure treatment adherence. In cases of non-compliance, it is crucial to counsel the patient effectively, employ various monitoring methods at different levels, and involve healthcare providers. Utilizing tools such as video-assisted techniques and medication event reminder monitors can significantly enhance treatment adherence. Additionally, prompt reporting and management of ADRs resulting from anti-tubercular therapy are essential for improving patient compliance. Adopting a positive and proactive approach among healthcare professionals will not only enhance treatment outcomes but also contribute to the eventual eradication of TB nationwide [11]. 

According to PMDT (programmatic management for drug-resistant TB) guidelines, whenever a patient admitted at the DR-TBC has serious adverse events (SAEs) to any of the anti-TB drugs, further management, withholding, or discontinuation of the drug is decided by the committee. Arrangement of drugs for the management of adverse events and timely and intensive monitoring are the essential components of the PMDT service. On-time, accurate, and proper reporting and analysis of adverse events are required as per program guidelines. Drugs for the management of SAEs should be made available to the patient free of cost, and proper training of healthcare workers, patient support, and management of the SAE at appropriate healthcare facilities will help to improve patient adherence to treatment, reduce mortality, and obtain better treatment outcome of the patient [11].

Patients require comprehensive support to navigate the challenges associated with TB and its treatment, including daily adherence hurdles, ADRs, indirect costs, and societal stigma. In many instances, due to various circumstances, only less potent and more toxic drugs remain available for treatment. Consequently, lengthy treatment regimens become necessary to ensure a cure without relapse. Extended regimens, particularly in cases of complex clinical conditions, such as advanced disease with a higher burden of bacilli and severe organ involvement, are often associated with heightened risks of toxicity. Factors contributing to this heightened risk include prolonged drug exposure, increased intolerance, adverse effects, and a greater potential for drug-drug interactions, particularly in critically ill patients [11,12].

The NTP should actively keep a close watch on drug safety to ensure proper care for patients, promptly report any negative reactions to the appropriate drug safety authority in the country, and contribute to shaping both national and global healthcare policies. Patients will require assistance in overcoming the challenges linked to TB and its treatment. Patients with comorbidity and ADR need to be managed separately. The TB treatment card may be changed to allow the tabulation of ADRs. It is also applicable for private practice [11,12].

Improving the quality of patient data necessitates the utilization of standardized variables, encompassing information on DST patterns, prescribed treatment, treatment outcomes, and ADRs. The gathering and utilization of such data hold significant importance for shaping future evidence-based recommendations, particularly in light of the scarcity of RCTs pertaining to the management of DR-TB [11]. If digital patient records are not currently in place, it is essential for program managers to consider their implementation, particularly for surveillance purposes and potentially for case management as well. In instances where patient records are already digital, adjustments may be necessary within the electronic recording and reporting system. This is to ensure the identification of individuals within specific MDR-TB regimen cohorts, such as those on shorter regimens or containing bedaquiline, as well as operational research subgroups. Additionally, certain options should be incorporated into the monitoring framework, such as the inclusion of clofazimine and the registration of ECG findings. It is critical for programs to meticulously and proactively maintain such data to contribute effectively to program evaluation and global policy-making. Additionally, electronic tools have the potential to improve the quantification of consumables. For instance, the volumes of medicine can be automatically calculated using QuanTB, a freely available application (app) that can be downloaded for enhanced efficiency [11].

The main focus of the recording and reporting activities in aDSM (adverse drug event surveillance and management) is to prioritize SAEs. However, it is crucial to manage any adverse event occurring during treatment administration, regardless of its relation to drug toxicity, to minimize harm to patients. MDR-TB treatment facilities may also monitor nonserious adverse events that hold clinical significance or are of special interest to the program as part of a more comprehensive aDSM approach. Within aDSM, adverse events are not only reported spontaneously but also actively elicited through a patient monitoring plan, which includes a set of questions and often involves various laboratory or clinical tests conducted at defined intervals before, during, and after treatment. For instance, the development of WHO consolidated guidelines greatly benefited from the insights derived from patient treatment experiences within programs [11].

Intra-dermal patch testing can be a safe and helpful diagnostic tool in the evaluation of cutaneous ADRs. Hypersensitivity reactions like urticaria and angioedema due to beta-lactams and cephalosporins like antimicrobials will be prevented [13].

## Conclusions

In this case, piperacillin-tazobactam-induced urticaria and angioedema are an immunoglobulin IgE-mediated hypersensitivity reaction. Also, the patient may develop this reaction with other antimicrobials due to cross-sensitivity, which should be preventable. Along with that, in this case, the patient was non-adherent to anti-tuberculosis therapy due to an ADR. So, a patient-centered approach including counseling, adherence support, close follow-up, and monitoring of ADRs was required. The successful management of ADRs and the promotion of treatment adherence are central to achieving the ultimate goal of eradicating TB in the country.
